# Supplementary material for: Predicting seasonal influenza using supermarket retail records
Source: PLoS Comput Biol. 2021 Jul 12;17(7):e1009087. doi: 10.1371/journal.pcbi.1009087 (PMC8297944; doi:10.1371/journal.pcbi.1009087)
Supplement: S1 Table — Performance indicators with respect to the Influnet ground truth for the sentinel basket forecast approach and the baselines (autoreg, Product-5) for individual seasons 2011/12, 2012/13, 2013/14 and 2014/15. (PDF) [file pcbi.1009087.s001.pdf]

S1 Table. Performance indicators for individual seasons. Performance indicators with respect to the Influnet ground truth for the sentinel basket forecast approach and the baselines (*autoreg*, *Product-5*) for individual seasons 2011/12, 2012/13, 2013/14 and 2014/15.

	**Pearson correlation***				**MAPE**				**RMSE**			
	1 week ahead	2 week ahead	3 week ahead	4 week ahead	1 week ahead	2 week ahead	3 week ahead	4 week ahead	1 week ahead	2 week ahead	3 week ahead	4 week ahead
**2011/12**												
autoreg	*0.95*	*0.87*	*0.85*	*0.83*	*11.04*	*22.82*	*29.53*	*38.03*	*0.92*	*1.59*	*1.73*	*1.91*
Product-5	*0.40*	*0.65*	*0.66*	*0.46*	*51.96*	*44.12*	*39.43*	*39.32*	*3.84*	*3.30*	*3.02*	*3.34*
Basket-1	**0.97**	0.92	**0.97**	**0.95**	**9.32**	17.35	**14.20**	16.66	**0.65**	1.25	**1.04**	**0.94**
Basket-5	**0.97**	**0.93**	0.93	0.93	11.78	**15.52**	14.83	**16.52**	0.71	**1.02**	1.09	1.15
**2012/13**												
autoreg	***0.97***	*0.90*	*0.90*	***0.91***	***8.12***	*14.21*	*17.24*	*24.79*	***0.85***	*1.46*	*1.61*	*1.81*
Product-5	*0.71*	*0.38*	*0.16*	*-0.03*	*35.99*	*48.04*	*48.97*	*58.00*	*2.32*	*3.35*	*4.34*	*4.77*
Basket-1	0.95	**0.94**	0.94	**0.91**	10.26	**12.23**	14.38	**17.61**	1.04	1.10	1.23	**1.45**
Basket-5	0.94	**0.94**	**0.95**	0.86	14.20	13.49	**12.23**	19.50	0.98	**1.03**	**0.92**	1.64
**2013/14**												
autoreg	***0.99***	*0.94*	*0.91*	*0.85*	*9.54*	*18.38*	*28.47*	*28.02*	*0.55*	*1.06*	*1.51*	*1.60*
Product-5	*0.23*	*0.06*	*-0.15*	*-0.24*	*40.40*	*38.36*	*50.48*	*68.26*	*2.41*	*2.37*	*2.51*	*2.98*
Basket-1	0.98	**0.97**	**0.95**	**0.91**	**7.37**	**8.63**	**9.70**	**19.55**	**0.52**	**0.61**	**0.68**	1.43
Basket-5	0.97	**0.97**	**0.95**	0.86	11.49	9.32	13.52	19.93	0.67	0.67	0.91	**1.40**
**2014/15**												
autoreg	***0.98***	*0.92*	*0.88*	*0.93*	*10.60*	*23.53*	*22.38*	*19.48*	*0.81*	*1.86*	*2.25*	*1.77*
Product-5	*0.87*	*0.68*	*0.42*	*0.24*	*38.87*	*36.77*	*37.78*	*38.27*	*2.83*	*3.16*	*3.45*	*3.62*
Basket-1	**0.98**	**0.97**	**0.96**	**0.96**	**8.12**	**8.32**	**10.98**	**12.98**	0.64	**0.91**	**0.82**	**1.04**
Basket-5	**0.98**	**0.97**	0.95	0.94	9.69	15.59	18.41	14.53	**0.59**	1.03	1.14	1.12

* for all coefficients p-value $<$ 0.01.
