# Supplementary material for: Predicting seasonal influenza using supermarket retail records
Source: PLoS Comput Biol. 2021 Jul 12;17(7):e1009087. doi: 10.1371/journal.pcbi.1009087 (PMC8297944; doi:10.1371/journal.pcbi.1009087)
Supplement: Attachment — Submitted filename: Response to reviewers.pdf [file pcbi.1009087.s002.pdf]

Response to reviewers

Reviewer #1:

In the current study Miliou et al present an influenza forecasting system based on combined supermarket purchases (baskets) in Italy. They compare their performance directly to autoregressive models and to autoregressive + individual item purchase performance, and indirectly to a prediction system using search queries (ref 57). They find their basket-based system systematically outperforms the other systems. This is a nice paper highlighting the use of a novel data source for pathogen surveillance. The problem I see is that it leaves me wanting more in terms of what the products are and further discussion of them. This should be straightforward for the authors to add.

Comments:

1. The authors have missed a citation on NDS (of which Vespignani is a coauthor):

Althouse & Scarpino, et al. "Enhancing disease surveillance with novel data streams: challenges and opportunities." EPJ Data Science 4.1 (2015): 1-8.

(Full disclosure: I am a coauthor of this paper) A citation would be good on line 3 or 31.

The citation has been added on line 31.

2. I want a lot more detail about what are in the baskets:

- What are common products?

- Are there any surprising products?

- How much do the baskets change season to season? (eg, how robust is this system?)

- Are there regional/country specific products which would make it difficult to translate to other regions/countries?

The discussion is too short and unsatisfying as is and could be a good place to go after these and other questions.

A subsection has been added (line 188) under the section Results, named “Explainability - Content of Sentinel Baskets”. In this subsection we discuss the content of the sentinel baskets and we address the questions asked from the reviewers.

Additionally, we updated the discussion to comment about the content of the sentinel baskets (lines 235-241 and lines 254-256).

3. Are the flu incidence data smoothed in figure 1?

The flu incidence data are not smoothed because Influnet provides the data pre-processed by the Italian National Institute of Health (Istituto Superiore di Sanità).

Reviewer #2: Predicting seasonal influenza using supermarket retail records

The article study the predictive power of supermarket retail data for influenza forecast in Italy. It is delivering a relatively straight forward message that the sales record of a selected baskets (termed “sentinel baskets” by the authors) can have information towards influenza situation.

The authors emphasize the the data proxy, rather than the forecast method. The authors used Support Vector Regression (SVR) to illustrate that using supermarket retail records, they can outperform the autoregressive model with historical ILI. This is an interesting article. I think overall the message is straightforward and supported by the results. Nevertheless, I have the following **comments**:

1. What are the most predictive retail subcategories (i.e., your sentinel products)? Are they mostly medical products? It is important to examine whether the products make intuitive sense.

A subsection has been added (line 188) under the section Results, named “Explainability - Content of Sentinel Baskets”. In this subsection we discuss the content of the sentinel baskets and we address the questions asked from the reviewers.

2. Why do you need to identify sentinel customers? Most big-data influenza prediction would aggregate the data over each individuals. I find it surprising that you need to identify the customers in this study. Isn’t it enough to use the aggregated purchase volume of the sentinel products? Your claim “we are interested in the purchases of these specific customers since those individuals would have a higher possibility to be either infected or close to an infected individual”, albeit sensible, seems to be tangent to this study, which is to predict ILI activity over entire population. It is also concerning from the privacy perspective.

In our actual predictions, we did not use those identified sentinel customers, but actually the aggregated signals. The necessity to identify the sentinel customers surges from the use of the Apriori algorithm that needs these detailed information to create less noisy and more robust proxy data, which are later aggregated for our predictions.

A paragraph has been added under the subsection Sentinel Customers to clarify this (lines 402-406).

3. How is your data correlate with other data sources? There are studies using pharmacy sales data. If one has access to pharmacy sales data, will your supermarket retail data add information on top of that? Why? The authors are welcomed to comment on this in the discussion.

Our retail dataset contains OTC (over-the-counter) drugs that are available without a prescription. Unfortunately, in our study, these products rarely correlate with influenza so they don’t occur among the sentinel products. A possible explanation is that when people get sick, they tend to visit the pharmacy directly and not the supermarket. Additionally, the OTC drugs are only available in the “IPERCOOP”, the largest stores of the supermarket chain, and as a result, their purchase trends are not necessarily representative of the whole dataset. Finally, very often, the drugs necessary to fight influenza symptoms are everyday drugs that normally every household owns and buys independently of the influenza occurrence.

A paragraph has been added under the section Discussion to clarify this (lines 242-250).
